# Effectiveness of enhanced cognitive behavioral therapy (CBT-E) in the treatment of anorexia nervosa: a prospective multidisciplinary study

**DOI:** 10.1186/s12888-016-1056-6

**Published:** 2016-10-05

**Authors:** Yngvild S. Danielsen, Guro Årdal Rekkedal, Stein Frostad, Ute Kessler

**Affiliations:** 1 Department of Clinical Psychology, University of Bergen, Christiesgt.12, Po.box. 7800, 5021 Bergen, Norway; 2 Division of Psychiatry, Haukeland University Hospital, Haukelandsveien.22, Po.box. 1400, 5021 Bergen, Norway

**Keywords:** Anorexia nervosa, Enhanced cognitive behavioral therapy, CBT-E, Treatment dropout, Gut microbiota, Genetics

## Abstract

**Background:**

Anorexia nervosa (AN) is a debilitating psychiatric disorder associated with a wide array of negative health complications and psychiatric comorbidity. Existing evidence for AN treatment in adults is weak, and no empirically supported treatment has been reliably established. The primary objective of this study is to gain knowledge about the effectiveness of enhanced cognitive behavioral therapy (CBT-E) for anorexia nervosa delivered in a public hospital setting. Baseline predictors of treatment outcome and dropout are studied. Furthermore, there will be collected blood and stool samples for a general biobank to be able to initiate research on possible pathophysiological mechanisms underlying AN.

**Methods:**

The study will assess the potency of outpatient CBT-E in a sample of patients suffering from AN (age >16) admitted to the Section for Eating Disorders at the Department for Psychosomatic Medicine, Haukeland University Hospital in Bergen, Norway. The study has a longitudinal design with five main assessment time points: before treatment, at 3 months, at the end of treatment, at 20 weeks, and at 12 months follow-up including biobank samples. A control group without an eating disorder will also be recruited.

**Discussion:**

Treatment research in a public hospital setting is important for gaining knowledge about the transportability of treatments evaluated in research clinics into ordinary clinical practice. Furthermore, biological material from the thoroughly described patient cohort will serve as a basis for further research on the pathophysiological mechanisms in AN.

**Trial registration:**

ClinicalTrials.gov Identifier: NCT02745067. Registered 14 April 2016. ﻿

## Background

### General overview

Anorexia nervosa (AN) is a debilitating psychiatric disorder characterized by abnormal eating behaviors, severe weight loss, intense fear of gaining weight or becoming fat (despite being underweight), and having a disturbed body image [1]. AN is associated with a wide array of negative health complications that cause suffering or even premature death in affected patients, as well as posing a heavy burden on public health-care services [2–4]. Psychiatric comorbidity is common with AN, with depression and anxiety disorders being the most prevalent [5], and there is a significantly increased risk of death by suicide [6]. The risk of mortality is reportedly sixfold higher in patients suffering from AN than in the general population [7], and a meta-analysis of 3006 patients admitted to hospital due to AN found that the 10-year mortality rate was 5.9 % [8].

Successfully treating AN is challenging. This condition is associated with a poor prognosis, including high rates of enduring illness and treatment dropout [9, 10]. Previous studies suggest that less than half of AN patients recover completely from the disorder. A substantial proportion of AN patients develop severe and enduring AN [11]. The adverse effects of AN combined with the high risk of severe and enduring AN indicate a substantial need for effective treatment options.

According to the two most widely influential treatment guidelines from the National Institute for Health and Clinical Excellence (NICE) [12] and the American Psychiatric Association [5], existing evidence for AN treatment in adults is weak, while Family-Based Treatment (FBT) is recommended for youths. The highest level of evidence for adult AN treatment in the NICE-guidelines is grade C, based on a consensus among experts rather than actual effectiveness as demonstrated in well-conducted clinical studies. The clinical consensus indicated that focal psychodynamic therapy, cognitive behavioral therapy (CBT), interpersonal psychotherapy, and cognitive analytic therapy are equally beneficial [5, 12].

The scarcity of research on treatments for AN might be partially due to several factors that complicate the ability to investigate this condition in well-designed studies, including its low prevalence, severity of medical complications, very high dropout rates, and the extended treatment duration required to successfully treat the disorder [13]. Halmi and colleagues [13] argued that it is necessary to study and address treatment dropout and refusal before further large randomized controlled trials (RCTs) of AN are initiated [13]. Fassino and colleagues argued [9] that avoiding treatment dropout is important, since those who drop out often have worse long-term prognoses, tend to be referred again at a later time when their problems are more severe, and their illness is more chronic and more prone to relapse [9]. Moreover, dropping out is expensive to society.

New treatment guidelines for AN are under development in Norway. There are five regional eating-disorder units in Norway, and they each apply somewhat different treatment approaches for AN, which reflects the difficulty of reaching an evidence-based consensus for treatment recommendations. Thus, there is a need for studies evaluating the treatment responses for different psychological AN treatments. Likewise, knowledge about which patients benefit from particular treatments, and predictors and reasons for treatment dropout is important for improving treatments, preventing treatment dropout, and making more-informed choices when tailoring treatments for individual AN patients.

### Evaluation of outpatient CBT-E as a standard treatment for adult AN patients in a public hospital

As discussed above, the existing evidence for AN treatment is weak and no empirically supported treatment has been reliably established for adults with AN [12, 14, 15]. There is therefore a need to document the effects of existing treatments for AN.

CBT-BN is currently the leading evidence-based treatment for bulimia nervosa (BN) [12]. The enhanced version of this treatment (CBT-E), developed during the last decade, has taken a “transdiagnostic approach” to eating disorders [16]. The CBT-E model is based on the assumption that common cognitive mechanisms, such as overevaluation of body shape and weight, underlie all eating disorders [17].

Fairburn and colleagues [18] investigated the outcomes in 99 adult patients with AN resulting from treatment with CBT-E [18]. The results showed that two-thirds of the patients completed the 40-week treatment intervention, with outcomes of clinically significant weight gains and significant reductions in eating-disorder pathology. The results further demonstrated that overall changes were well maintained after a 60-week follow-up period [18]. These findings are in line with another study suggesting that CBT-E is an effective treatment for adolescents with AN [19]. A recent study from Germany (the ANTOP study) compared CBT-E and focal psychodynamic therapy with optimized treatment as usual, and found significant weight gains in all treatment groups, but no significant differences in the effects on body mass index (BMI) and eating-disorder pathology between the treatment approaches [20]. These studies suggest that CBT-E could be a useful treatment approach for adult AN.

Our study will evaluate the use of CBT-E as a standard treatment for AN in the regional eating-disorder unit of a public hospital. Based on a survey of eating-disorder professionals, Wallace and von Ranson [21] argued that there is a disconnection between research and practice in eating-disorder treatment [21]. They further claimed that when empirically supported interventions are implemented in practice, they tend to become embedded within more eclectic treatment approaches and are not used in their original forms as evaluated in research clinics. Our study is one of the first to aim at using the CBT-E protocol as a standard noneclectic treatment for all AN patients able to undergo psychotherapy in a regional eating-disorder unit at a public hospital.

### Baseline predictors of treatment outcome and dropout, and dropout analysis

High dropout rates have been regarded as a major problem in AN treatment research since they make it difficult to conduct RCTs. This further implies that a relatively large proportion of patients are not able to benefit from current AN treatments. Few previous studies have investigated the characteristics of those patients who do not benefit from AN treatment and their reported reasons for dropping out. Moreover, the predictors of treatment outcome and dropout have to our knowledge not been studied in CBT-E treatment for AN. Such knowledge could act as an important guide for identifying those patients who might need other treatment approaches or how treatments may be improved or tailored for different subgroups of patients.

The rate of treatment dropout is generally reported to vary between 29 % to 73 % in outpatient samples of AN patients [9]. Dropout rates of 36 % and 37 % have been reported for CBT-E treatments of AN [18, 19]. The ANTOP study found dropout rates of 19 % for CBT-E and 34 % for focal dynamic therapy in AN patients [20], suggesting that dropout rates from CBT-E are not higher than those of other treatments for AN, but that they are still significant.

Even though dropout is a major problem with AN treatments in general, and knowledge about the predictors and reasons for dropping out are important in planning and improving interventions, the findings of the few studies investigating this have been ambiguous, and they have mainly focused on personality factors and aspects of the eating disorder [9]. One study examining predictors of dropping out from CBT-E treatment among a mixed group of eating-disorder patients found that treatment dropout was predicted by a history of very low weight, avoidance of affect, and a longer time on waiting lists before starting treatment [22]. Surprisingly few studies have investigated how sociodemographic factors influence treatment outcomes, with one recent review concluding that such data are more or less nonexistent [23].

In terms of clinical experiences of AN, higher symptom severity, longer duration of illness, lower baseline BMI, higher number of prior treatment attempts and dropouts, higher age and degree of psychiatric symptoms and comorbidity are often considered to make treatment and recovery more difficult. The present study will examine these baseline characteristics as well as sociodemographic factors as predictors of treatment outcomes and dropout. The findings should constitute new and valuable clinical evidence for guiding which groups of patients are most likely to benefit from CBT-E treatment for AN.

Subjective experiences of the patients are also important when evaluating the reasons for treatment dropout and the suitability of a treatment. Possible reasons for treatment dropout include (1) lack of motivation, (2) feeling able to manage on your own and not needing further treatment, (3) dissatisfaction with the treatment model, (4) not feeling capable of doing the tasks required, (5) lack of good alliance with the therapist, (6) not experiencing satisfactory results from the treatment, and (7) having other competing activities, work difficulties, family problems, or other health problems. To our knowledge there are no previous reports on patient evaluations of the reasons for the premature termination of AN treatment.

### Process factors, therapeutic alliance, and early patient engagement in treatment as predictors of outcome and dropout

Most previous studies of predictors of treatment outcome and dropout from AN treatment have focused on characteristics of the patients. Therefore, the factors related to the treatment process itself warrant investigation [22].

An early response to treatment might reflect patient engagement in therapy and readiness to change, which might be predictive of the eventual treatment outcome or dropout [24]. In CBT-E [17], patient adherence to the behavioral goals of the treatment during the first 4 weeks is suggested to be predictive of the overall treatment outcome; however, this is a hypothesis that requires further scientifically testing. Two studies of different treatments of AN found that adequate weight gain over the first 4 weeks significantly predicted retention in the treatment program [25, 26]. The time before starting to regain weight during treatment and the rate of weight gain might be strong predictors of treatment outcome and retention. In addition, a reduction of binge-eating episodes and vomiting during the first weeks of treatment and the establishment of regular meals seem to be significant prognostic factors [17, 24, 27]. The present study will acquire weekly data on BMI, number of days with regular eating, and the numbers of weekly binge-eating and purging episodes. This information might be useful for clarifying to what extent an early change in CBT-E treatment is predictive of the overall treatment outcome.

The therapeutic alliance is one of the factors found to be related to symptom changes and treatment outcome in psychological treatment research [28, 29]. Establishing an early strong alliance in the treatment of AN has traditionally been considered difficult because these patients often deny the presence of the disorder. Further, AN patients usually have severely reduced cognitive function due to malnutrition, and most patients are ambivalent to treatment [30]. However, while some studies in contrary to this assumptions have found quite a strong therapeutic alliance in treatments for AN [31, 32], few studies have examined the nature of the relationship between therapeutic alliance and symptom change in AN [31–34]. One study found the therapeutic alliance in CBT treatment for AN to be relatively strong, but interestingly weight gain was found to predict a stronger alliance while a strong early alliance did not predict weight gain [31].

### Psychiatric comorbidity

Psychiatric comorbidity is common in AN, with depression and anxiety disorders being the most prevalent conditions. The American Psychiatric Association has reported lifetime prevalence rates for major depressive disorder in individuals with eating disorders of between 50 % and 75 % [5]. Previous studies that have compared comorbidity between AN and mood disorders have produced widely varying findings, with lifetime prevalence rates of between 31 % and 88.9 % [35]. However, these studies have not always addressed the difference between psychological symptoms related to AN and comorbid psychiatric disorders. This is particularly interesting because there is a clinical consensus that symptoms of depression and anxiety with AN are partially linked to malnutrition, and are symptoms of starvation [5]. The widely cited starvation study conducted by Keys and colleagues in 1950 indicated that malnutrition causes anxiety and depressive symptoms [36]. However, few subsequent studies have shed further light on the link between malnutrition and these psychological symptoms [37]. A review of seven studies investigating the relationship between BMI and depression/anxiety symptoms found general decreases in depression and anxiety symptoms associated with weight gain after treatment [37]. From this perspective, depression and anxiety represent interesting secondary outcome measures in the treatment of AN.

### Physical activity

High-level physical activity, excessive exercise, and compulsive exercise play a central role in the eating disorder pathology of many patients with AN, and these might have been part of the pathogenesis of their disorder [38]. Several terms are used to describe the nature of physical activity that is considered a maintaining factor in AN, some focusing on the quantitative dimension of physical activity while others are related to the more-subjective experience of the compulsiveness of physical activity [39]. This compulsiveness has been suggested to be more connected to eating disorders than the excessiveness of the exercising itself [40]. Further, the regulation of negative emotions and compulsivity can maintain excessive levels of physical activity [41]. Dalle Grave and colleagues [39] found that compulsive exercise was performed by 80 % of patients with restrictive AN and by 43.3 % of patients with purging AN.

Excessive exercise and high-level physical activity have not been widely studied in AN, and there are neither established terminologies nor criteria for their use in definitions or measurements [39, 42]. Most studies rely on self-report measures of physical activity, and so new studies using objective measures are warranted [42]. Alberti and colleagues [43] compared a self-report measure and an objective measure of physical activity in AN patients, which revealed significant underreporting of physical activity level in this population.

There is a need for more knowledge about the impact of high-level physical activity and compulsive exercise on the treatment outcome and maintenance in AN. Dalle Grave and colleagues [39] found that a lower amount of exercise during the preceding 4 weeks predicted an improved eating-disorder symptomatology at the treatment follow-up. Two other studies found excessive exercise to be related to longer treatment duration [44], a more-enduring eating-disorder pattern, and early relapse after treatment [45]. These studies indicate that excessive exercise/high level physical activity/compulsive exercise is important to address and manage in the treatment of AN [46].

### Biobank

Despite the rapidly increasing knowledge about several aspects of the pathophysiology of AN, there is still no unifying theory for its etiology. AN is a multifactorial disease, and environmental, social, psychological, genetic, bacterial, and endocrine factors are thought to influence its pathophysiology [47, 48]. Acknowledging this, the present study will establish a biobank (blood and stool samples) to be able to initiate research on possible pathophysiological mechanisms that play a role in the pathophysiology of AN. Specifically, we will focus on the gut microbiota as well as genetic and biochemical/immunological markers. The longitudinal design of this study and the inclusion of a healthy control group are significant strengths that will contribute to a better understanding of AN pathophysiology, which could have implications for treatment efficacy.

## Research questions

Based on the literature discussed above, this study will address and attempt to answer the following main research questions:

**Research question 1: Evaluation of outpatient CBT-E as a standard treatment for AN**

Do adult AN patients provided with CBT-E treatment in a Norwegian outpatient setting show favorable pre-post changes, and are these sustained at 12 months after ending treatment? We expect changes to occur in the following areas: weight gain and eating-disorder pathology, depressive symptoms and depression, anxiety, general psychological impairment, and health-related quality of life.

**Research question 2: Baseline predictors of treatment outcome and dropout, and dropout analysis**

- Do baseline BMI, duration of illness, number of prior treatment attempts, number of prior dropouts, time on waiting lists, socioeconomic status, work status, social network support, level of depression, anxiety, presence of other psychiatric diagnoses (as assessed using the Mini International Neuropsychiatric Interview [MINI]), and general psychological symptomatology predict treatment outcome or dropout?
- Which factors do patients who drop out from CBT-E treatment report as reasons for ending treatment prematurely? This will be an explorative study and we would expect patients who drop out to report different reasons when interviewed, such as lack of motivation, feeling satisfied with their current situation, fear of change, dislike of the treatment method, difficulties in performing the tasks required, poor relationship with the therapist, and presence of other illnesses or other competing activities.

**Research question 3: Therapeutic alliance, and early patient engagement in treatment as predictors of pre-post outcome and dropout**

Do early (4 weeks) behavioral changes, adherence to treatment, and therapeutic alliances predict the pre-post outcome and dropout in CBT-E treatment for AN?

**Research question 4:** To what extent is there a link between weight and depressive symptoms; does change in BMI predict change in depressive symptoms?

**Research question 5: Physical activity**

- Does the physical activity level as measured by accelerometers differ between AN patients and matched normal-weight healthy controls without an eating disorder?
- Is there a change in excessive or compulsive exercising (pretreatment versus posttreatment)?
- Does high-level physical activity and compulsive exercise at baseline predict a worse treatment outcome as determined by BMI and the EDE-Q and Clinical Impairment Assessment Questionnaire (CIA)?
- Does a higher level of physical activity at the end of treatment predict relapse or higher scores on EDE-Q at 12 months after ending treatment?

## Methods/Design

### Study design

The current project is an open, longitudinal treatment study undertaken in an ordinary clinical setting at a regional eating-disorder unit (Section for Eating Disorders, SED). All patients will receive a minimum of 40 sessions of outpatient CBT-E that cover the different treatment sessions described in the CBT-E manual for underweight patients [17], which is the standard treatment for AN at our unit. A control group without eating disorders will also be recruited.

### Patient sample

All patients who agree to receive treatment for AN at the SED will be asked to participate in the study. Patients are referred to the clinic by general practitioners and secondary (specialist) health-care services. Written informed consent will be obtained after the patients have received written and oral descriptions about the study. During the past 2 years, 96 AN patients were offered treatment at the SED. We aim to include 100 patients in the current study. Analyses of statistical power showed that the proposed sample sizes in the different studies are sufficient.

The study will include patients aged >16 years who are suffering from AN, as diagnosed based on the DSM-5 (Diagnostic and Statistical Manual of Mental Disorders, Fifth Edition) [1] and confirmed with a clinical examination. Patients offered treatment at the SED have either at least one unsuccessful treatment attempt in a secondary health-care service unit or severe AN that is evaluated as not manageable in a secondary health-care service unit. Participation in the study will be offered to patients younger than 18 years if they do not fulfill the criteria for receiving FBT for AN [49]. Patients will be excluded from participation if it is deemed unsafe to manage them on an outpatient basis or have psychiatric comorbidity (as evaluated with MINI [50]) that precludes a focused eating-disorder treatment, such as psychosis or drug abuse. Patients who are not available to participate during the requested treatment period will also be excluded.

In line with clinical practice, patients will be withdrawn from outpatient treatment if they are unable to participate actively in the treatment program according to the judgement of the clinician, based on not attending sessions (more than three consecutive treatment sessions without giving notice) or not engaging in using the treatment tools (e.g. not self-monitoring).

Treatment at the SED is offered in a stepped care model, whereby patients participating fully in outpatient treatment but still not achieving the necessary changes will be offered a more-intensive treatment option (Intensive CBT-E) as inpatients or in day care. These patients will be included as a separate group in the analyses.

### Control group

A healthy control group (consisting of 30-35 persons) will be recruited through advertisements and matched to the patient group in terms of age, gender, and length of education. In order to control for longitudinal changes, the control group will be tested twice, with an interval of 1 year. The control group will provide activity measures, blood and stool samples, and complete the EDE-Q.

### The intervention: CBT-E

The theory that underlies CBT-E is focusing on the mechanisms that maintain eating-disorder psychopathology rather than the processes that initially led to the development of the disorder (see a thorough overview of CBT-E [17]). CBT-E has the following four distinct treatment stages:

1. The main priority in stage 1 is to engage the patient in treatment and behavioral changes. This stage includes creating a personalized formulation, establishing self-monitoring and in-session weighing, and introducing and establishing a regular eating pattern (at this point the therapist is not concerned about the quantity and composition of meals) and providing psychoeducation (on weight, weight changes, and eating disorders in general).
2. Stage 2 is a brief transitional stage in which the therapist and the patient together review the treatment process, which should identify patients at risk of a poor outcome.
3. Stage 3 is the main part of the treatment, whose aim is to address the core mechanisms that are maintaining the patient’s eating disorder (underweight, overevaluation of body shape and weight, and control over eating, dietary restraint and restriction, and event- and mood-triggered changes in eating).
4. The aim in stage 4 is to ensure that the implemented changes are long-lasting, and to minimize the risk of relapse [17].

Sessions are provided twice weekly in stage 1, weekly in the subsequent stages, and biweekly toward the end of the intervention. One of the main goals of CBT-E treatment is to help the patient remain in the treatment program until it is completed. The treatment may end before it is completed if either the patient or the therapist decides to stop. If a patient wants to stop the treatment, the therapist will try to explore the reasons while focusing on the expectation that completing the treatment will produce a good outcome. If the patient then still wants to stop the treatment, it will be ended. The therapist will stop the treatment when it is concluded that the treatment is not working according to the plan. The therapist will always try to resolve these problems with the patient. If there are no acceptable solutions, the therapist will end the treatment before it is completed. When this happens the therapist will always try to make the patient understand why treatment is ending and also try to obtain mutual agreement that there are no other options.

### Intensive CBT-E

Intensive CBT-E treatment is performed on an inpatient basis and involves the same procedures and strategies as outpatient CBT-E, but it is more intensive. The method is described by Riccardo Dalle Grave [51]. Each patient will work within a multidisciplinary team composed of a physician, a psychologist, a physiotherapist, and nurses. The use of assisted eating is one of the main differences from outpatient CBT-E [52]. The nurses and the rest of the staff at the unit are trained in CBT-E in order to be able to help the patient prepare for the meals and to help the patient use the tools of cognitive behavior therapy for eating disorders to identify and address the maintaining mechanisms of the eating disorders. Standard duration of the inpatient CBT-E is 13 weeks. Inpatient therapy can be prolonged if BMI is below 16 or if the patient has additional problems to address (e.g. comorbidity). After inpatient therapy the patient can have four to six weeks of day treatment before outpatient CBT-E is conducted. Expected weight gain during inpatient CBT-E is 1,0 – 1,5 kg per week until normal weight is established.

### Concomitant treatment

All patients are advised to take the following standard dietary supplements daily: two omega-3 capsules, a 500-mg calcium tablet, and a multivitamin tablet. Patients with severe clinical depression will be treated with antidepressants. Most patients will have depressive symptoms secondary to long standing underweight. This depression is usually moderate and is not treated with antidepressants. In case of depression that makes CBT-E ineffective, treatment is suspended until pharmacological antidepressive treatment is established and stable. Sometimes a decision to start antidepressants can be made before psychotherapy starts, but it might be necessary to try psychotherapy and observe whether the clinical depression makes CBT-E impossible to perform. If there are not specific contraindications, fluoxetine 20 mg a day is started, after one week dosage is increased to 40 mg and after another week fluoxetine is increased to 60 mg a day. The patient is assessed weekly for side effects. If 60 mg is well tolerated, antidepressant effects are usually observed two weeks after having started with 60 mg. If the patient experience unacceptable side effects, fluoxetine is stopped and other antidepressant is started, usually escitalopram. If fluoxetine is well tolerated but sufficient antidepressive effects are not observed olanzapine 5 mg is added.

If a patient receives additional psychological or somatic treatment for comorbid conditions, this will be recorded.

### The therapists

Each patient will be treated by a single therapist. All of the therapists are trained in CBT-E through individual weekly supervision by an experienced on-site CBT-E therapist in their first year of delivering CBT-E. In addition, to ensure that treatment is correctly implemented and delivered equally to all patients, weekly supervision meetings attended by all of the therapists will be held throughout the study. Each of the cases will be individually discussed at these 2-hour weekly meetings. All of the therapists have been participating in monthly video-conference supervisions performed by the international collaborators Chris Fairburn and Riccardo Dalle Grave, and this supervision will continue throughout the study.

### Assessment and measurement points

The present study has five main assessment time points: at baseline (before treatment), at 3 months after starting treatment, at the end of treatment, at 20 weeks after ending treatment, and at 12 months after ending treatment. In addition, certain clinical measures will be recorded on a weekly basis, and early behavioral changes, treatment adherence, and therapeutic alliances will be measured at 4 weeks into treatment. Table 1 provides an overview of all of the study measures and their measurement time points. Most of the questionnaires will be completed using an Internet-based assessment platform, called CheckWare; the security of patient anonymity of this Web-based solution is approved by the Western Norway Regional Health Authority. The forms can be completed at home or on a computer in the clinic. The CheckWare program is creating e-mail reminders for the therapists when measurements are upcoming or delayed. The therapists schedule a time for the assessment at a computer in the clinic prior to a treatment session. The Web-based program immediately creates scores and diagrams based on the assessments that are reviewed by the therapist and patient together in the following session. Further, in the initial stages of the project there will be monthly meetings between research personnel and the therapists where feedback is given and potential problems with the data collection is discussed.

**Table 1:** Overview of the study measures and their measurement time points

	Measure	Baseline before treatment	Weekly during treatment	4 weeks after starting treatment	3 months after starting treatment	End of treatment	20 weeks after ending treatment	12 months after ending treatment
Initial subject and disease characteristics	Sociodemographic background information and illness history	x						
	BMI	x	x	x	x	x	x	x
Eating-disorder diagnosis ﻿and comorbidity	EDE-Q	x			x	x	x	x
	Other mental-health diagnoses	x			x	x	x	x
	MINI 6.0.0	x				x		x
	Other somatic ICD-10 diagnoses	x			x	x	x	x
Eating behaviors	Days with regular eating		x					
	Weekly binge-editing episodes		x					
	Weekly purging episodes		x					
	Current medications	x			x	x	x	x
Blood sampling	Potassium	x			x	x	x	x
	Venous base excess	x			x	x	x	x
	Estradiol	x				x		x
	FSH	x				x		x
Blood sampling in biobank	Genetic and other relevant biomarkers	x			x	x	x	x
	BMD (DEXA)	x				x		x
	Work status	x			x	x	x	x
Psychometric measures								
	CIA	x			x	x	x	x
	BDI	x			x	x	x	x
	BAI	x			x	x	x	x
	RAND-36	x				x		x
	SCL-90-R	x				x		x
Treatment parameters	Measure of early adherence			x				
	WAI-S			x		x		
	Type of treatment (CBT-E or Intensive CBT-E)	x			x	x	x	x
	Active, finished, or dropped out	x			x	x	x	x
	Parallel psychological treatment (other diagnose)	x			x	x	x	x
Activity measures	Accelerometry	x				x		x
	Daily log of physical activity (over 7 days)	x				x		x
	EED	x				x		x
	CET	x				x		x
Stool sample in biobank	Bacteriological measures	x			x	x		x
	IBS self-report	x			x	x		x

### Demographic and clinical characteristics

Data will be collected on age, gender, socioeconomic status, work status, marital status, children, living situation, duration of illness, prior treatment, lowest weight, highest weight, smoking habits, and prior treatment attempts.

### Mini International Neuropsychiatric Interview

The MINI is a short structured psychiatric diagnostic interview for disorders included in the DSM-IV (Diagnostic and Statistical Manual of Mental Disorders, Fourth Edition) and ICD-10 (10th revision of the International Statistical Classification of Diseases and Related Health Problems) [50]. The present study will use the Norwegian version of the MINI (version 6.0.0) [53], which has been validated in clinical settings and has shown satisfactory psychometric properties [54].This Norwegian version of the MINI has not been updated for DSM-5.

### Primary outcome measure

#### Eating Disorder Examination Questionnaire

The EDE-Q [55] is a 28-item self-report instrument designed to measure eating pathology. A recent review systematically studied the psychometric properties of the EDE-Q, and concluded that the instrument is reliable and valid for assessing eating-disorder symptoms [56]. The EDQ-E consists of four subscales: restraint, eating concern, shape concern and weight concern. The summation of the scores on all subscales produce a global EDE-Q score.

We will use a Norwegian translation that has Norwegian norms, good reliability, and expected correlations with BMI in a community sample [57, 58].

### Secondary outcome measures

#### Clinical Impairment Assessment Questionnaire

The CIA is a 16-item self-report questionnaire designed to measure the severity of psychosocial impairment associated with eating disorders. The original version of the scale has been shown to have good reliability, validity, and sensitivity to change [59]. We use a Norwegian translation with Norwegian community norms and which has been shown to be significantly correlated with EDE-Q scores (*r* = 0.83, *p* < 0.01) and BMI (*r* = 0.31, *p* < 0.01) in a community sample [60].

#### Body weight and BMI

BMI will be calculated in the standard manner, as weight in kilograms divided by the square of the height in meters. Weight will be measured using a beam-balance scale and height will be measured using a wall-mounted height board.

#### Beck Depression Inventory

The Beck Depression Inventory-II (BDI-II) is a 21-item self-report inventory designed to measure the degree of depressive symptoms in adolescents (>13 years) and adults [61]. Its psychometrical properties have generally been found to be satisfactory [62]. A recent review found good internal consistency (around 0.9) and good test–retest reliability (ranging from 0.73 to 0.96) [63].

#### Beck Anxiety Inventory

The BAI (Beck Anxiety Inventory) is a 21-item self-report inventory designed to measure the severity of anxiety in adolescents and adults. Its psychometrical properties have been found to be satisfactory, with high internal consistency (α = 0.92) and test–retest reliability [r(81) = 0.75] [64].

#### RAND 36-item Health Survey

The RAND 36-item Health Survey (RAND-36) [65] is a generic multidimensional self-report questionnaire. It consists of 36 questions that assess the 8 health concepts. The 36 questions are also distributed as the Short-Form Health Survey (SF-36) [66] by the Medical Outcomes Trust. The RAND-36/SF-36 is the most widely used measure of health-related quality of life [67]. The RAND-36 has shown satisfactory psychometrical properties [68].

#### The Symptom Checklist-90-Revised

The Symptom Checklist-90-Revised (SCL-90-R) is a 90-item self-report symptom inventory designed to measure psychological symptoms and psychological distress [69].

### Other measures

For a complete overview over all measures in all the different parts of the study, see Table 1.

#### Working Alliance Inventory–Short Form

The WAI-S (Working Alliance Inventory–Short Form) is a 12-item self-report measure of the general alliance in therapy and three different specific aspects of the therapeutic alliance: bonds, goals, and tasks. This measure has both patient and therapist versions, and it has been found to exhibit acceptable validity and interrater reliability [70–72]. This instrument will be administered at 4 weeks into treatment and at the end of treatment.

#### Checklist for dropout interviews

The checklist for dropout interviews will include specific items asking patients whether or not they agree with statements about reasons for ending treatment, including motivation, dislike of the treatment form, being satisfied with their current status, feeling able to continue on their own, not being satisfied with the treatment results, finding the tasks during treatment too difficult, having problems relating to the therapist, having difficulties due to the presence of other illnesses, and having practical problems related to work, studies, family relationships, and finances. The interview will also ask a general question about other reasons, and it will be conducted by a study secretary via telephone within a few weeks of the treatment ending prematurely.

#### Evaluation of early adherence, behavioral changes, and self-monitoring

The CBT-E treatment model includes performing a standardized evaluation of progress at 4 weeks into treatment using the CBT-E stage-2 evaluation form [17]. Different aspects of patient behavioral adherence to treatment are evaluated on the following 3-point scale: not going well, going reasonably well, and going well. The rated behaviors are attending sessions, being on time, self-monitoring, not weighing yourself at home, reading the provided psychoeducational materials, eating regular meals, not eating between meals, and prioritizing treatment.

Each day during CBT-E treatments, the patients complete self-monitoring sheets reporting the times of meals, what they have been eating and drinking, episodes of binge eating and vomiting, and other compensatory strategies. These are reviewed in the sessions with the therapist. In addition, they weigh themselves during the sessions. This constitutes the weekly acquisition of data on BMI, days with regular eating, and numbers of weekly binge-eating and purging episodes.

#### Accelerometers

Physical activity and movement will be measured using wrist-worn accelerometers (AW2 actigraph, Philips/Respironics). The accelerometer will be worn for 7 consecutive days and nights at the following measurement time points: baseline, at the end of treatment, and at 12 months after ending treatment. This device measures both the frequency and magnitude of movement. The use of accelerometers for measuring physical activity and sleep has been validated [73, 74]. During the same period the participants will keep a daily log of their physical activity.

#### The Exercise and Eating Disorders

The Exercise and Eating Disorders questionnaire (EED) [40] is a new self-report questionnaire developed to obtain more-detailed information about exercise regimes and disturbances among patients with eating disorders. This questionnaire focuses on compulsive exercise as a symptom of an eating disorder. The EED consists of 22 items scored on a 6-point scale from 0 (never) to 5 (always). This measure will be applied at the same time points as when accelerometry is performed.

#### Compulsive Exercise Test

The Compulsive Exercise Test (CET) is a self-report questionnaire developed to assess the primary factors influencing the maintenance of excessive exercise. The CET consists of 24 items with 5 subscales: avoidance and rule-driven behavior, weight-control exercise, mood improvement, lack of exercise enjoyment, and exercise rigidity. Preliminary testing for the psychometric properties of the CET has shown promising results [41]. This measure will be applied at the same time points as when accelerometry is performed.

#### Blood samples and biobank

We will obtain a venous blood sample from each patient at five time points, as indicated in Table 1.

Further, we will obtain additional blood samples for later studies of (epi)genetic and other biological markers of interest in AN. Those samples will be stored as whole blood, serum, and on an RNA stabilization medium in a biobank established for the current study. The biobank will be colocalized with an existing biobank (“ADHD in Norway”; the responsible professor is Jan Haavik, University of Bergen) and using the existing infrastructure for registration, storage, and alarms.

We expect further progress in this field before and during the study, and so there might be changes in the most-suitable analyzing methods for both DNA and RNA and the other biomarkers.

#### Stool samples

Stool samples will be collected from AN patients and healthy controls at four time points. Samples collected at home or in the hospital will be frozen and stored. Several methods are suitable for the characterization of the fecal microbiota. Because of the expected further advances in molecular microbiological approaches the methods will be specified later.

#### Power analyses

All power analyses were conducted with G*Power software (version 3.1.7) [75]. A large effect size is expected for the study treatments (*d* = 0.8). Alpha was set to 0.05 and the power was set to 0.80. The coefficient for the correlation between measurement points was set to 0.50. The results showed that 12 subjects would be needed to detect a significant effect of time. In terms of dropouts, Pr(Y = 1∣X = 1) H0 (which signifies the probability of dropping out when all predictors/covariates are set to their mean values) is set to 0.25. For a two-tailed odds ratio of 2.0, an alpha of 0.05, and a power of 0.80, a sample size of 100 is needed when the R^2^ values of other predictors are set to 0. Linear regression of the treatment outcome with an expected regression coefficient of *d* = 0.20 (two-tailed) and with alpha set to 0.05 and the power set to 0.80 indicated that 81 subjects are needed.

#### Study setting

The SED is a regional unit for the most severely ill eating-disorder patients in Western Norway. This unit serves a population of 1 million inhabitants. Since 2007, all therapists at the SED have contributed to comprehensive data collection as part of internal quality control. As a follow-up and extension of this work, a Regional Research Database (Western Norway) for eating disorders has been designed, which is located at the SED. This database has been approved by the Norwegian Data Inspectorate, and data collection was commenced in January 2016. The present study will use data from this database in addition to measures specifically added to test the hypotheses of this study. The present study has been designed in close collaboration with the therapists at the SED in order to ensure that it will be possible for them to perform the data collection. The process assessments included in the present study are already embedded in the clinic and are applied in clinical practice.

## Discussion

Conducting studies in an ordinary health care setting poses both challenges and advantages. An advantage is the possibility to test how treatments evaluated in research clinics translate into an ordinary public hospital setting.

A challenge when implementing studies in an ordinary clinical setting is to secure the quality of the data collection and avoid missing data. The therapists have a busy schedule and assessments for the study need to be embedded into their daily routines and be experienced as meaningful for both therapists and patients. To secure this a detailed protocol for the data collection is written in co-operation with the therapists. Further, the therapists have been involved in designing the study through several meetings.

At 12-months follow-up the participants in the treatment study are compensated for their travel expenses for coming to the clinic to avoid this being a barrier for participating. The healthy weight controls likewise get their expenses covered as well as receiving a reward of 500 NOK (about 40 Euros).

Another aspect of implementing a treatment study into clinical practice is the question of patients receiving different treatments based on entering the study or not; and the need to follow national clinical guidelines for treatment. This makes a randomized controlled trial difficult to conduct in this setting. A waitlist condition also would prove ethically difficult because of the severity of the illness. Accordingly, in this study all patients receive the same treatment irrespective of study participation and there is no waiting-list or comparison group for the treatment condition. This constitutes a weakness when drawing conclusions based on treatment response.

To secure patient safety in cases of withdrawing from the study the routines are as follows: For patients deciding to leave the study, treatment continues. If the patient decides to end treatment pre-maturely they are referred back to their primary GP or to other relevant treatment. There is always an invitation to return to the eating disorder treatment at a later point of time if this is relevant and the patient wants to work in treatment again.

The longitudinal design of this study and the inclusion of a healthy control group are significant strengths that will contribute to a better understanding of AN pathophysiology, which could have implications for treatment efficacy.

## Abbreviations

AN: Anorexia nervosa
BAI: Beck Anxiety Inventory
BDI-II: Beck Depression Inventory-II
BMD: Bone mineral density
BMI: Body mass index
BN: Bulimia nervosa
CBT: Cognitive behavioral therapy
CBT-E: Enhanced cognitive behavioral therapy
CET: Compulsive Exercise Test
CIA: Clinical Impairment Assessment Questionnaire
DEXA: Dual-energy X-ray absorptiometry
DSM: Diagnostic and Statistical Manual of Mental Disorders
EDE-Q: Eating Disorder Examination Questionnaire
EED: Exercise and Eating Disorders questionnaire
FBT: Family-based treatment
FSH: Follicle-stimulating hormone
IBS: Irritable bowel syndrome
IBS-SSS: IBS Symptom Severity Scale
ICD-10: 10th revision of the International Statistical Classification of Diseases and Related Health Problems
MDD: Major depressive disorder
MINI: Mini International Neuropsychiatric Interview
NICE: National Institute for Health and Clinical Excellence
RAND-36: RAND 36-item Health Survey
RCTs: Randomized controlled trials
SCL-90-R: Symptom Checklist-90-Revised
SED: Section for Eating Disorders
SF-36: Short-Form Health Survey
WAI-S: Working Alliance Inventory–Short Form
